# Tuning the Proton‐Coupled Electron‐Transfer Rate by Ligand Modification in Catalyst–Dye Supramolecular Complexes for Photocatalytic Water Splitting

**DOI:** 10.1002/cssc.202001863

**Published:** 2020-09-15

**Authors:** Yang, Huub J. M. de Groot, Francesco Buda

**Affiliations:** ^1^ Leiden Institute of Chemistry Leiden University Einsteinweg 55 2300 RA Leiden (The Netherlands

**Keywords:** *ab initio* calculations, dye-sensitized photoanode, proton-coupled electron transfer, vibronic coupling, water splitting

## Abstract

In view of the considerably high activation energy barrier of the O−O bond formation photocatalytic step in water oxidation, it is essential to understand if and how nonadiabatic factors can accelerate the proton‐coupled electron transfer (PCET) rate in this process to find rational design strategies facilitating this step. Herein, constrained *ab initio* molecular dynamics simulations are performed to investigate this rate‐limiting step in a series of catalyst‐dye supramolecular complexes functionalized with different alkyl groups on the catalyst component. These structural modifications lead to tunable thermodynamic driving forces, PCET rates, and vibronic coupling with specific resonant torsional modes. These results reveal that such resonant coupling between electronic and nuclear motions contributes to crossing catalytic barriers in PCET reactions by enabling semiclassical coherent conversion of a reactant into a product. Our results provide insight on how to engineer efficient catalyst‐dye supramolecular complexes by functionalization with steric substituents for high‐performance dye‐sensitized photoelectrochemical cells.

## Graphical abstract

**Tuning couples**: Tunable resonant coupling between electronic and nuclear motions is achieved by structural modifications at the interface between the water oxidation catalyst and the dye in a series of supramolecular complexes for photocatalytic water splitting. These nonadiabatic factors lead to a considerable increase of several orders of magnitude in the reaction rate for the O−O bond formation step.

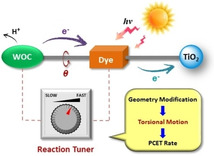

## Introduction

Solar‐driven water splitting via dye‐sensitized photoelectrochemical cell (DS‐PEC) devices is an area of rapid technological growth, and is considered to be a promising scalable, affordable and sustainable technology for direct solar‐to‐fuel conversion to produce strategically valuable and storable hydrogen, or hydrocarbons from CO_2_.^[1]^ Decentralized PEC offers intrinsic advantages since the integration of the PV and electrocatalytic steps in one device operating at low current density reduces overpotential and concentration losses compared to centralized electrolysis driven by PV.^[2]^ For one complete water splitting cycle in DS‐PECs, four photons are absorbed at the photoanode, generating holes on the light‐harvesting dye that should provide sufficient driving force for the four‐proton/four‐electron water oxidation half‐reaction catalyzed by a water oxidation catalyst (WOC). The four photo‐generated electrons migrate to the (photo)cathode to be consumed for the hydrogen production or for CO_2_ reduction.^[3]^ Despite the effort in the development of novel DS‐PECs, which have been improved either in the photoelectrodes^[4]^ or in the ion‐exchange membrane,^[5]^ the overall yield of the water oxidation half‐reaction is limited. In particular the O−O bond formation step represents a thermodynamic and kinetic bottleneck for productive forward electron transfer.^[6]^ This leads to low yield, often less than 20 %, due to charge recombination losses at the dye‐electrode interface.^[7]^

Proton‐coupled electron transfer (PCET)^[8]^ plays an essential role in the photocatalytic four‐proton/four‐electron oxidation of water. Proper assembly of the components in the WOC‐dye supramolecular complex provides channels for PCET steps in which the electron and proton are transferred in different directions and the dye is regenerated to its initial state.[^6^, ^9^] The critical O−O bond formation process with mononuclear catalysts is found to be the most challenging and the rate‐limiting step in catalytic water oxidation.^[10]^ Significant rate enhancement has been achieved either by modifying the ligand of the WOC or by tuning the solvent environment, in which computational studies act as a powerful technique.^[11]^

In catalysis, electrons are generally considered to be in equilibrium with their atomic surrounding, and reactions are thought to proceed adiabatically over catalytic barriers. While recent analysis of PCET reactions acknowledge the importance of nonadiabatic terms connecting electronic states, these are usually treated as probabilistic events for the conversion of reactants into products in the context of nonadiabatic transition state theory.^[12]^ However, when reactant and product levels cross due to molecular vibrations, resonant vibronic coupling can be established over an avoided crossing that provides a fast deterministic semiclassical coherent channel from the reactant to the product output, in particular for asymmetric systems that evolve along a torsional degree of freedom.^[6]^ While we have found convincing evidence that resonant coupling is important for energy transfer and separation of charges,^[13]^ the purpose of this study is to investigate the possibility for resonant coupling at the crossing of the reactant and product states for the O−O bond formation in water oxidation, and if this offers an attractive chemical engineering principle to achieve near‐unity yield in photochemical water oxidation.

In the context of PCET reactions in artificial photosynthesis, the photocatalytic water splitting cycle in a WOC‐dye supramolecular complex [(cy)Ru^II^bpy(H_2_O)]^2+^‐NDI (cy=*p*‐cymene, bpy=2,2′‐bipyridine, NDI=2,6‐diethoxy‐1,4,5,8‐diimide‐naphthalene) has recently been systematically investigated *in silico*, providing the driving force and the energy barrier of each PCET step by DFT‐based molecular dynamics (DFT‐MD) simulations.[^6^, ^14^] The computed energy barrier (ΔG*=15.9 kcal mol^−1^) and corresponding reaction rate (*k*=15.7 s^−1^) confirm that the third catalytic PCET step involving the O−O bond formation is indeed the kinetic bottleneck of the entire catalytic water oxidation half‐reaction, which would increase the possibility of charge recombination and thus lower the quantum yield.[^6^, ^15^]

**Scheme 1:**
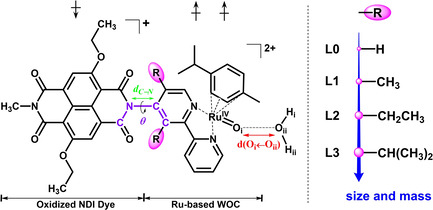
Schematic structure of complexes **L0‐L3** (^2^([Ru^IV^=O]_*i*_^2+^−NDI^+.^), *i*=0−3) after the photooxidation of NDI dye together with the attacking water molecule in the vicinity of Ru center: The dihedral angle *θ* and the C−N bond studied in this work are indicated in purple and green, respectively. The spin multiplicity 2S+1=2 for a net spin S=1/2 in this case corresponds to two unpaired α electrons (↑) localized on the catalyst and one unpaired β electron (↓) on the oxidized NDI^+.^. The red double‐sided arrow indicates the reaction coordinate d(O_i_←O_ii_) considered in the constrained MD simulations.

**Scheme 2:**
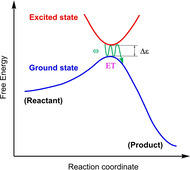
Schematic illustration of the resonant coupling between electronic and nuclear motions in the investigated system. Δ*ϵ* stands for the excitation energy around the transition state. *ω* represents the vibrational frequency of the torsional angle *θ*.

In this work we explore the possibility of enhancing the rate of this critical PCET step in the WOC‐dye complex [(cy)Ru^II^bpy(H_2_O)]^2+^−NDI by modifying the bipyridine ligand that is covalently bound to the NDI dye (see Scheme 1). Specifically, a series of alkyl groups varying in size and mass were introduced in the bpy residue near the C−N bond connecting the WOC and the NDI dye (**L0‐L3** in Scheme 1). The rationale for this choice is to affect the torsional motion at the interface between the WOC and the dye in order to match the associated nuclear frequency (ω) to the resonance condition for the electron transfer process (ω≈Δ*ϵ*, see Scheme 2).^[13b]^ This is inspired by the correlation between the torsional motion and the electron dynamics observed in our previous investigation of the catalytic cycle.^[6]^

Understanding the impact of coherent coupling between electron and nuclear motions in catalytic reactions, such as the rate of this PCET reaction in the WOC‐dye complex, is of particular interest and great significance.^[16]^ With this aim, we perform DFT‐MD simulations following the Car‐Parrinello approach to obtain accurate predictions of the activation energy barrier.^[17]^ We show how the electron transfer is coherently coupled to a specific torsional motion, and how the reaction rate of this catalytic PCET reaction in the WOC‐dye complex (^2^([Ru^IV^=O]^2+^‐NDI^+.^), see Scheme 1) is affected by the ligand modifications.

## Results and Discussion

### Geometry optimization of the WOC‐dye complexes

**Figure 1:**
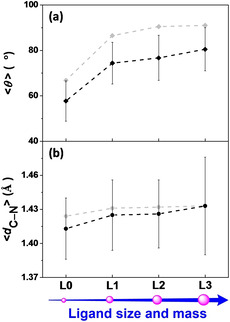
a) Time‐averaged dihedral angle (<*θ*> in °), b) C−N bond length (<*d*_C‐N_> in Å), and corresponding standard deviations of complexes **L0‐L3** during the constrained MD simulations in explicit water solvation. For comparison, the dihedral angles (*θ* in °) and C−N bond lengths (*d*_C‐N_ in Å) of complexes **L0‐L3** after geometry optimization with the ADF program using OPBE functional, the TZP basis set, and implicit solvation (COSMO) are indicated in grey.

The initial geometry of the photo‐oxidized WOC‐dye complexes **L0‐L3** is optimized at the density functional theory (DFT) level employing the OPBE exchange‐correlation functional^[18]^ and the TZP (triple‐ζ polarized) Slater‐type basis set in implicit solvation (COSMO) with the Amsterdam Density Functional (ADF) software package^[19]^ (see the Supporting Information S1 for more computational details).^[14]^ The increase in size and mass of the ligand R leads to an elongation of the C−N bond (*d* ^0^_C‐N_) linking the WOC and dye components and to an increase of the dihedral angle (*θ* ^0^) around the C−N bond from **L0** to **L3**, due to the steric hindrance from bulky substituents (see Figure 1, grey scatters and Table S1 in Supporting Information, section S2). The initial geometry will determine the sign of the dihedral angle as steric hindrance prevents the system to flip from a positive value of *θ* ^0^ to an equivalent geometry with an opposite value, effectively breaking this symmetry.

**Figure 2:**
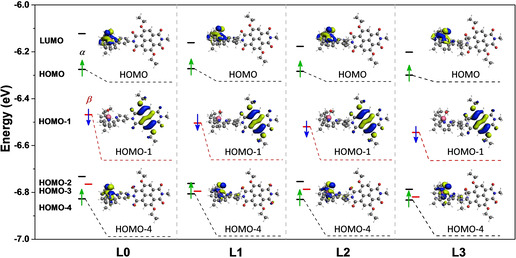
Selected frontier molecular orbitals of complexes **L0‐L3** (^2^([Ru^IV^=O]_*i*_^2+^‐NDI^+.^), *i*=0−3) after photooxidation of the NDI dye computed with the ADF program using the OPBE functional and the TZP basis set. The continuum solvation model (COSMO) is used to describe the water environment. Energy levels are indicated in black for spin α and in red for spin β orbitals, respectively. Only the unpaired electrons are explicitly indicated by vertical arrows (green for an unpaired electron localized on the catalyst and blue for an unpaired electron on the oxidized NDI^+.^) and the corresponding isosurface representation is shown in the inset. See Table S2 for the molecular energy levels.

Using the optimized geometry of the photo‐oxidized WOC‐dye complexes, we analyze the electronic structure and in particular the frontier molecular orbitals that play a crucial role in the PCET step and in regenerating the ground state of the dye. Figure 2 illustrates the frontier molecular orbital energy levels together with an isosurface corresponding to the singly occupied molecular orbitals (SOMOs) of complexes **L0‐L3**. The corresponding energy levels are also listed in Table S2 (see Supporting Information, section S3). For all these complexes, the SOMO localized on the oxidized NDI^+.^ (SOMO dye) is always lower in energy than the HOMO of the supramolecular complex, which is localized on the ruthenium catalyst (SOMO WOC). Moreover, the energy difference between the SOMO dye and the SOMO WOC (Δ*E* in Table S2) is found to systematically increase as the size and mass of the ligand R increases from complex **L0** (Δ*E*=0.193 eV) to **L3** (Δ*E*=0.245 eV). This result suggests an increasingly larger driving force for electron transfer from the ruthenium catalyst to the oxidized NDI dye due to the geometrical distortion induced by the steric hindrance from bulky substituents.

### Equilibration of the WOC‐dye complexes in explicit solvent model

An accurate description of the PCET reaction and corresponding free energy profile requires an explicit inclusion of the water environment as it is crucially involved in the reaction process.[^6^, ^11^, ^15^] Therefore, an orthorhombic box of dimensions 25.1×17.7×14.4 Å^3^ with periodic boundary conditions containing the WOC‐dye solute **L0‐L3** together with 162 explicit water molecules is used in the DFT‐MD simulations performed with the CPMD program.^[20]^ The DFT electronic structure is determined by using the OPBE exchange‐correlation functional,^[18]^ GTH pseudopotentials for the ruthenium transition metal^[21]^ and dispersion‐corrected pseudopotentials (DCACP) for the remaining atoms,^[22]^ together with a plane wave cutoff of 70 Ry (see Supporting Information, section S1 for more computational details). An initial free DFT‐MD simulation of 0.6 ps at room temperature is performed for each [WOC]^2+^‐dye solvated system to equilibrate the solvation environment. Prior to this DFT‐MD run, the systems have been already pre‐equilibrated with classical force field (see Supporting Information, section S1.2).

In a previous work^[14]^ we have demonstrated that upon photoexcitation the NDI is able to inject an electron at a dye‐sensitized TiO_2_ semiconductor surface on a time scale of ∼1 ps. It is therefore reasonable to assume in the following analysis that the system is already in its oxidized form [WOC]^2+^‐dye^+.^ at the beginning of the simulation for this catalytic PCET step driven by the complexes **L0‐L3**. The photooxidation of the NDI dye is mimicked by removing one electron from the simulation box after the initial equilibration simulation for each system considered. Subsequently, the oxidized state is further equilibrated for another 0.6 ps at room temperature. We show in Figure S1 (see Supporting Information, section S4) that the running average of the Kohn‐Sham energy reaches a stable value even within this relatively short MD timescale of ∼0.6 ps. Notice that during all the MD simulations after the photooxidation of the NDI, we only focus on the most favorable reaction route recently reported with a total electron spin angular momentum S=1/2 . This is assumed to be conserved along the reaction coordinate since the O−O bond formation is thermodynamically unfavorable for the S=^3^/_2_ case.^[6]^ When the spin density is tracked along the equilibration MD simulation for the solvated [WOC]^2+^‐dye^+.^ systems, two unpaired α electrons (↑) are observed to localize on the catalyst and one unpaired β electron (↓) on the NDI dye in all the systems (see insets in Figure S2). Thermal fluctuations of the total spin density localized on the NDI dye, of the dihedral angle (*θ*) and C−N bond length (*d*_C−N_) along this free MD trajectory are also collected in Figure S2 (see Supporting Information, section S4).

### Constrained MD simulations of the O−O bond formation step

After this equilibration simulation, the constrained MD approach combined with thermodynamic integration is then employed to estimate the free energy profile of the third catalytic water oxidation step (see the redox couple in Equation (1), where H_2_O_sol_ and H^+^_sol_ represent the solvated attacking water molecule and proton respectively):

(1) $${}^{2}\left(\left[\mathrm{Ru}{}^{\mathrm{IV}}=O\right]{}_{i}{}^{2+}\text{-}\mathrm{NDI}{}^{+}{}^{•}\right)+H{}_{2}O{}_{\mathrm{sol}}\vec{\leftarrow }{}^{2}\left(\left[\mathrm{Ru}{}^{\mathrm{III}}-\mathrm{OOH}\right]{}_{i}{}^{2+}\text{-}\mathrm{NDI}\right)+H{}^{+}{}_{\mathrm{sol}},\;\left(i=0-3\right)$$

In Equation (1) the total spin multiplicity 2S+1=2 is maintained over the WOC‐dye system, with doubly charged WOC catalytic intermediates on both sides of the redox couple. Hence one electronic spin quantum is internally transferred from the NDI to the WOC, while one unit of charge is released into the solvent environment in the form of a proton. The use of constrained MD is appropriate here since this reaction is a rare event on the typical DFT‐MD simulation time scale.^[23]^ The constrained reaction coordinate is the distance between the oxygen atoms O_i_ and O_ii_ indicated by the red double‐sided arrow in Scheme 1 (see Supporting Information, section S1 for more computational details). In similar computational work on O−O bond formation, metadynamics simulations have been used as an alternative enhanced sampling method.^[24]^ In particular, in a very recent work in addition to the O−O distance, a second collective variable has been included to keep track of the proton transfer.^[24d]^ In our investigation, we didn't introduce additional constraints for the proton transfer to avoid a bias on the proton acceptor.

**Figure 3:**
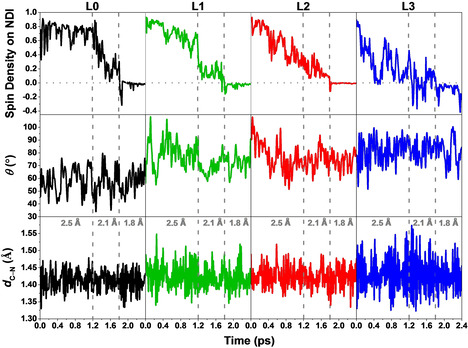
Spin density integrated over half of the simulation box including the NDI dye, time evolution of the dihedral angle (*θ*) and C−N bond length (*d*_C−N_) of complexes **L0‐L3** along the constrained MD trajectories. An integrated spin density value of 1 corresponds to one unpaired β electron (↓). The value of the constrained reaction coordinate d(O_i_←O_ii_) in the MD simulations is noted in grey.

In order to explore the correlation between the electronic and nuclear motions in these WOC‐dye complexes, the variation of the spin density on the NDI dye together with the time evolution of the dihedral angle (*θ*) and C−N bond length (*d*_C−N_) for complexes **L0‐L3** along the constrained MD trajectories are collected in Figure 3. The time‐averaged dihedral angle (<*θ*>), C−N bond length (<*d*_C‐N_>), and corresponding standard deviations for all complexes **L0‐L3** during the constrained MD simulations are presented in Table S1 and Figure 1 for a quantitative comparison. According to the results of our DFT‐MD simulations, the introduction of a ligand R larger in size and mass than hydrogen in complexes **L1**−**L3** gives rise to an increasing dihedral angle (74.7–80.5°), and longer C−N bond (1.426–1.433 Å) as well as larger fluctuations during the dynamics compared to those of complex **L0** (57.7°, 1.413 Å). The trend of the computed time average <*θ*> and <*d*_C‐N_> when gradually enlarging the size and mass of ligand R from **L0** to **L3** is consistent with the static DFT results (see Table S1 and black scatters in Figure 1).

The electron transfer is affected by the dynamic structure and starts already in the constrained MD with the reaction coordinate value d(O_i_←O_ii_)=2.5 Å in **L1**−**L3**, while it occurs only after further shortening the reaction coordinate d(O_i_←O_ii_) to 2.1 Å in the case of **L0** (Figure 3, top panels). In particular for **L3** there is oscillatory behaviour. Initially the electron transfer from the WOC to the photoinduced hole on the oxidized NDI occurs very rapidly already with d(O_i_←O_ii_)=2.5 Å, as it can be visualized by the spin density on the NDI going to zero in about 0.4 ps. This rapid event is then followed by a partial back transfer and pronounced fluctuations. The electron keeps transferring back and forth between the WOC and dye even when we further continue the constrained 2.5 Å MD simulation for another ∼0.6 ps (see Supporting Information, section S5). In Figure S4 in Supporting Information, we show that the running average of the Lagrangian multiplier reaches a stable value within the constrained 2.5 Å MD timescale of ∼1.2 ps for complexes **L0‐L3** although large fluctuations on spin density could still be observed at the end of this simulation.

In all cases, the electron transfer is completed at d(O_i_←O_ii_)=1.8 Å with a stable integrated spin density value of 0 on the NDI (see also Supporting Information, section S7, where we show a longer constrained simulation with d(O_i_←O_ii_)=1.8 Å for **L3**), corresponding to the final state with one unpaired α electron (↑) localized on the catalyst and no unpaired electron on the NDI dye, which is regenerated to its initial ground state. The proton transfer from the attacking water molecule to the solvent only occurs during this constrained 1.8 Å simulation when the electron transfer is completed. In particular, the proton H_i_ diffuses into the solvent bulk via a “chain” of hydrogen‐bonded water molecules, which can be well described by the Grotthuss mechanism^[25]^ (see Supporting Information, section S8). This mechanism has been already observed in our previous works.[^6^, ^15^] The reaction coordinate d(O_i_←O_ii_) is then further shortened to 1.6 Å to better explore the complete free energy profile along this reaction pathway and no back reaction occurs (see Supporting Information, section S9). More importantly, no back‐transfer of either an electron or a proton is observed even after the release of the constraint between O_i_ and O_ii_ at the end of the 1.6 Å simulation, confirming the stability of the final product [see Equation (1)] after the O−O bond formation (see Supporting Information, section S10).

The facilitation of electron transfer by ligand modification can be partially attributed to the larger driving force for bulkier substituents from **L0** to **L3** as discussed earlier in terms of molecular orbital energies (see Figure 2). However, another important factor could be a resonant coupling between electronic and nuclear motion that will be discussed further in a next section.

### Free energy profile and reaction rate estimation

**Figure 4:**
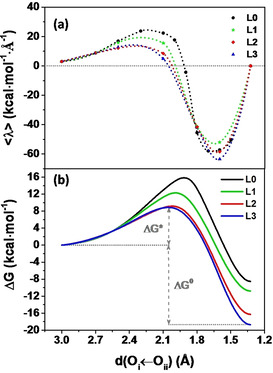
a) Time‐averaged constraint force represented by the Lagrangian multiplier <λ> computed for each constrained MD simulation as a function of the reaction coordinate d(O_i_←O_ii_) for complexes **L0‐L3**. The Akima splines (100 points) is used to interpolate the mean forces. b) Free energy profile along the reaction coordinate d(O_i_←O_ii_) computed from thermodynamic integration for complexes **L0‐L3**. The time‐averaged constraint forces and associated free energy profile for **L0** are taken from Ref. [6] for comparison.

Based on all the constrained DFT‐MD simulations performed, the free energy profile along the reaction coordinate d(O_i_←O_ii_) of **L0‐L3** can be computed using the Bluemoon ensemble approach.[^23^, ^26^] This will allow for a quantitative evaluation of the effect of the ligand modification on the rate enhancement for this catalytic water oxidation step. The time‐averaged forces associated with the applied constraints, the interpolation of the time‐averaged mean forces used for this analysis, and the corresponding free energy profile obtained via thermodynamic integration of **L0‐L3** are presented in Figure 4 (see Supporting Information, section S1.3 for computational details and section S11 for error bar of each time‐averaged mean force).

It is apparent from Figure 4b that the obtained activation energy barrier for O−O bond formation decreases systematically as an effect of the increasing steric hindrance by substitution of ligand R in the sequence **L0**→**L1**→**L2**→**L3**. In addition, following the same sequence, the transition state occurs earlier, *i. e*., at larger values of d(O_i_←O_ii_) along the reaction coordinate. The key thermodynamic parameters extracted from the free energy profile of **L0‐L3** for this water oxidation step are summarized in Table 1 (see Supporting Information, section S1.5 for more details). The energy difference between the SOMO dye and the SOMO WOC (Δ*E*) of complexes **L0‐L3** after the photooxidation of the NDI dye is also included in Table 1 for comparison. In particular the calculated activation free energy barriers ΔG*, 9.2 kcal mol^−1^ (∼0.40 eV) and 8.9 kcal mol^−1^ (∼0.39 eV) for **L2** and **L3** respectively, are dramatically lowered almost by half in comparison with that of **L0** (15.9 kcal mol^−1^ (∼0.69 eV)), indicating that this catalytic process is significantly facilitated by the changes in electronic and structural dynamics resulting from the ligand modification. It should be emphasized that the photooxidation of the NDI dye makes this reaction exothermic in all considered cases. However, following the order **L0**→**L1**→**L2**→**L3** the driving force becomes stronger, since Δ*G* ^0^ increases systematically from −8.5 to −18.7 kcal mol^−1^. Table 1 shows a clear inverse correlation between the driving force and the activation free energy, which is consistent with Hammond's postulate.^[27]^ Interestingly, the variation in Δ*G* ^0^ is much larger than the increase in the static molecular orbital energy difference Δ*E* (see Table 1), pointing to the importance of dynamical (entropic) effects.

**Table 1:** Computed activation energy barrier (ΔG*), thermodynamic driving force (Δ*G* ^0^), and estimated reaction rate (*k*) of the third PCET step involving the O−O bond formation for the complexes **L0‐L3**, together with the energy difference between SOMO dye and SOMO WOC (Δ*E*) of complexes **L0‐L3** obtained with static DFT calculations.

WOC‐dye complex	ΔG* [kcal mol^−1^]	Δ*G* ^0^ [kcal mol^−1^]	Δ*E* [kcal mol^−1^]	*k* [s^−1^]
**L0** ^[a]^	15.9	−8.5	−4.5	15.7
**L1**	12.3	−10.8	−5.1	6.6×10^3^
**L2**	9.2	−16.3	−5.5	1.2×10^6^
**L3**	8.9	−18.7	−5.6	2.0×10^6^

[a] The results for **L0** are taken from Ref. [6].

The computed activation free energy barrier ΔG* of **L0‐L3** can be used to determine the reaction rate (*k*) according to transition state theory.^[28]^ The predicted reaction rate reported in Table 1 validates the Bluemoon constrained MD approach and shows an enhancement of up to 5 orders of magnitude from **L0** (15.7 s^−1^) to **L3** (∼2.0×10^6^ s^−1^) as an effect of the ligand modification. To address now the crucial question about the origin of this very large effect on the activation energy barrier and hence the reaction rate, we look into the coupling between the electronic and the nuclear motion.

### Coupling between electronic and nuclear motions

**Figure 5:**
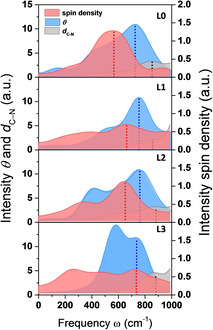
Frequency spectrum associated to the electron transfer (red) and the vibrational density of states (VDOS) of the dihedral angle (*θ*, blue) and the C−N bond length (*d*_C−N_, grey) for complexes **L0‐L3** extracted from the constrained 2.5 and 2.1 Å MD trajectories corresponding to Figure 3.

To resolve possible resonant coupling between the electron transfer process and specific nuclear motions and how this affects the reaction rate of this catalytic water oxidation step, it is convenient to analyze the DFT‐MD trajectories in the frequency domain.^[13a]^ Thus, the Fourier transform of the velocity autocorrelation function is calculated for the time evolution of the spin density and for the thermal fluctuations of *θ* and *d*_C−N_ along the constrained MD trajectories corresponding to Figure 3, in which the electron transfer (ET) takes place. The Fourier transform of the electron‐transfer time evolution as well as the vibrational density of states (VDOS) of *θ* and *d*_C−N_ are presented in Figure 5. We herein only focus on the range of 0–1000 cm^−1^ since no distinct overlap between nuclear and electronic spectra is found at frequencies higher than 1000 cm^−1^ (see Supporting Information, section S12).

In Figure 5 (top panel), the starting complex **L0** shows for each spectrum one main peak located at 566, 726, and 854 cm^−1^ for spin density (red), *θ* (blue), and *d*_C−N_ (grey), respectively. The modification of ligand R in complex **L1**–**L3** induces the appearance of a second peak at lower frequencies in the spectrum of the ET and *θ*, while the spectrum of *d*_C−N_ stays essentially unchanged. Noticeably, the main peak of the ET spectrum (red dotted lines in Figure 5) is clearly shifted to higher frequency going from **L0** to **L1**–**L3**, leading to increasing overlap with the higher‐frequency peak in the spectrum of *θ* (see blue dotted lines in Figure 5). In particular, these two peaks are both centered at around 735 cm^−1^ for the **L3** complex (see Figure 5). These results strongly suggest that the ligand modifications induce resonance due to converging timescales of the nuclear vibration of the torsional angle *θ* and the electronic motion of the charge transfer. The C−N stretching mode instead appears to have less overlap with the ET frequency spectrum. Considering the accelerated reaction rate obtained by modification of the ligand R (see Table 1), one is tempted to conclude that the resonance condition achieved between the ET frequencies and the VDOS of *θ* plays a dominant role in accelerating a catalytic reaction between different electronic states. In other words, the ligand modifications increase the nonadiabatic coupling between reactant and product states, which contributes to the acceleration of the reaction rate in a semiclassical, coherent conversion process that is deterministic instead of probabilistic.

In order to further validate the relevance of vibronic coupling in determining the reaction rate, an additional constrained DFT‐MD simulation was carried out for **L3** after the photooxidation of NDI with d(O_i_←O_ii_)=2.5 Å and with fixed *θ*=91°, as this is the dihedral angle obtained from the DFT geometry optimization of **L3** (see Figure 1). The time evolution of the spin density localized on the NDI shows that the electron transfer from the WOC to the oxidized NDI dye is strongly inhibited when fixing the torsional angle *θ* (see Supporting Information, section S13), which highlights the crucial role of this particular nuclear motion in facilitating the ET process. One can also notice that the value of *θ* extracted from the optimized geometries is about the same for the initial intermediate and for the final product after this catalytic step (see Supporting Information, section S14). Therefore, by fixing the value of *θ* we are not preventing a specific change in the dihedral angle from the initial to the final value, but we are only removing the vibrational motion of *θ*, and thus the coupling with the electronic charge fluctuations.

Our further argument supporting the idea of an increased nonadiabatic coupling driving catalysis going from **L0** to **L3** is based on the calculation of the excitation energies near the transition states. The results from TDDFT calculations (see Supporting Information, section S15) show that the energy difference between the ground state and the first excited state, which corresponds to the charge transfer state, decreases from ∼1.8 kcal mol^−1^ for **L0** to ∼1.3 kcal mol^−1^ for **L3**. This energy difference is comparable to the energy of the characteristic torsional frequencies (735 cm^−1^=2.1 kcal mol^−1^) shown in Figure 5.

## Conclusions

We have shown that by changing the mass and size of the ligand R at the interface between the water oxidation catalyst (WOC)and the dye, one can accelerate the proton‐coupled electron transfer (PCET) reaction step associated with the O−O bond formation by several order of magnitudes. The structural modifications modulate not only the value of the dihedral angle at the WOC‐dye linkage but also the electronic structure of the supramolecular complexes and the characteristic frequencies associated with the electron transfer dynamics and the torsional motion around this link. A similar strategy has been very recently used by synthetically modifying an iron chromophore to interfere with specific atomic motions and resulting in a dramatically different charge transfer lifetime.^[29]^ This frequency tuning leads to a resonance condition that increases the coupling between electronic and nuclear motions and facilitates the electron transfer step from the WOC to the oxidized dye in the region of the crossing of reactant and product states, in a process previously denoted nonadiabatic conversion by adiabatic passage (NCAP).[^2^, ^13b^, ^30^] The computed free energy profiles for this PCET reaction show a considerable decrease in activation energy and increase in the driving force. We expect that the in‐depth insight into the acceleration of this specific catalytic water oxidation step provides a general and rational engineering approach for the improvement of the performance of dye‐sensitized photoelectrochemical cell (DS‐PEC) devices from a structural design perspective, which can also be achieved by modifying other ligands around the connecting region or replacing the linker between WOC and dye.

## Conflict of interest

The authors declare no conflict of interest.

## Supporting information

As a service to our authors and readers, this journal provides supporting information supplied by the authors. Such materials are peer reviewed and may be re‐organized for online delivery, but are not copy‐edited or typeset. Technical support issues arising from supporting information (other than missing files) should be addressed to the authors.

Supplementary
