# Supplementary material for: Tuning the Proton‐Coupled Electron‐Transfer Rate by Ligand Modification in Catalyst–Dye Supramolecular Complexes for Photocatalytic Water Splitting
Source: ChemSusChem. 2020 Sep 15;14(1):479–86. doi: 10.1002/cssc.202001863 (PMC7821158; doi:10.1002/cssc.202001863)
Supplement: Supplementary file 1 — Supplementary [file CSSC-14-479-s001.pdf]

ChemSusChem

Supporting Information

Tuning the Proton-Coupled Electron-Transfer Rate by Ligand Modification in Catalyst–Dye Supramolecular Complexes for Photocatalytic Water Splitting

Yang Shao,* Huub J. M. de Groot, and Francesco Buda*© 2020 The Authors. Published by Wiley-VCH GmbH. This is an open access article under the terms of the Creative Commons Attribution License, which permits use, distribution and reproduction in any medium, provided the original work is properly cited.

Table of Contents

S1. Computational Details……S3
S1.1 Geometry Optimization at DFT Level……S3
S1.2 Simulation Box……S3
S1.3 Effect of Periodic Boundary Conditions……S3
S1.4 Free Energy Profile……S3
S1.5 Reaction Rate……S3
S2. Geometrical parameters……S4
S3. Molecular orbital and electronic structure……S4
S4. Equilibration of the WOC-dye complexes in explicit solvent model……S5
S5. Continued constrained 2.5 Å MD simulation for **L1**……S6
S6 Running average of the constraint force as a function of time……S6
S7. Continued constrained 1.8 Å MD simulation for **L3**……S7
S8. Proton transfer for **L0** – **L3** at d(O_i←O_{ii}) = 1.8 Å……S8
S9. Constrained 1.6 Å MD simulation……S9
S10. O–O bond formation during the free MD simulation……S10
S11. Time-averaged constraint force and standard deviation……S11
S12. Fourier transform and vibrational density of states (VDOS)……S12
S13. Constrained MD simulation with fixed dihedral angle θ for **L3**……S13
S14. Geometry optimization after PCET……S13
S15. TDDFT calculation of the excitation energies near the transition state……S14
References……S14

Experimental Procedures

S1. Computational Details

S1.1 Geometry optimization at DFT level

The OPBE exchange-correlation functional[1] and the TZP (triple-ζ polarized) Slater-type basis set[2] were employed in the geometry optimization of the initial and final states of WOC−dye complexes **L0** – **L3**. The OPBE functional has shown to be accurate in describing transition-metal complexes, including Ru-based WOCs.[3] In the geometry optimization, the continuous solvation model (COSMO[4]) for water was used. These calculations are performed with the Amsterdam Density Functional (ADF) software package.[5]

S1.2 Simulation box

To obtain a realistic description of the catalytic reaction step, the solvent was explicitly introduced in the simulations. The solvent environment for the Car−Parrinello MD (CPMD) simulations was generated using Discovery Studio 2.5.[6] The solvent was equilibrated for 0.2 ns using the TIP3P model implemented in the CHARMM force field and CFF partial charge parameters at 300 K,[7] while the $[WOC]^{2+}$−dye complex was kept fixed. The volume was then adjusted using constant pressure for 0.2 ns, after which the system was further allowed to evolve with constant volume for 2 ns. Periodic boundary conditions are applied with a time step of δt = 5 a.u. (1 a.u. = 0.0242 fs).

S1.3 Effect of periodic boundary conditions

Periodic boundary conditions (pbc) are applied in our simulations. In plane wave based AIMD simulations the periodic boundary conditions introduce a spurious Coulomb interaction for charged systems due to the image charges. The effect of pbc for charged systems can be important when dealing with isolated molecules/clusters in the simulation box. However, because of the quite large simulation box (25.1 × 17.7 × 14.4 $Å^3$) used and the fact that the MD simulation box contains 162 water molecules that will strongly screen the spurious Coulomb interaction. The spurious effect of periodic charges is estimated to be rather small (~0.01 eV). We can therefore conclude that the error introduced by the pbc does not affect significantly the conclusions of our work.

S1.4 Free energy profile

To estimate the activation free energy barrier of the catalytic reaction step involving the O–O bond formation that are unlikely to occur spontaneously during the typical *ab initio* molecular dynamics (AIMD) simulation time scale, constrained MD and the so-called Blue-Moon approach were employed as a rare event simulation technique.[8] The reaction coordinate (in this case the distance between two oxygen atoms O_i and O_{ii}, $d(O_i \leftarrow O_{ii})$, as shown in Scheme 1) is constrained to a series of fixed values x in range of 2.5 − 1.6 Å after the initial equilibrium simulation and subsequent photooxidation of NDI along this reaction pathway. A time-averaged constraint force $\langle\lambda\rangle_x$ for each value of the reaction coordinate x is obtained, which should be equal to zero at an equilibrium or transition state. Considering what we learned from our previous work that the reaction coordinate $d(O_i \leftarrow O_{ii})$ = 3.0 Å and 1.325 Å are quite close to the initial/final equilibrium state and far from the transition state, we therefore assume that the modification of ligand R has minor effect on the position close to the initial/final equilibrium state and all the complexes **L0** – **L3** share the same value for the constraint forces at the reaction coordinate $d(O_i \leftarrow O_{ii})$ = 3.0 Å, 2.7 Å, and 1.325 Å.[9] The activation free energy barrier for this catalytic step is then established by interpolating the mean forces with a 100-point Akima splines function and integrating the signed forces $\langle\lambda\rangle_x$ along the reaction path.[10] Trajectory analysis and visualization for the CPMD output were carried out using VMD.[11]

S1.5 Reaction rate

The computed activation free energy barrier can be used to evaluate to what extent the geometry modification accelerates the rate of the third water oxidation step involving the O−O bond formation. According to the transition state theory[12], the reaction rate (k) determined by the activation energy barrier (ΔG^*) can be expressed as

$$k = \frac{k_B T}{h} \cdot e^{-\frac{\Delta G^*}{RT}} \qquad (1)$$

Where ΔG^* represents the activation free energy barrier, k_B, h, R and T are the Boltzmann constant, the Planck constant, the universal gas constant and thermodynamic temperature, respectively. One should keep in mind that in the DFT-based MD simulations protons are treated classically and thus proton tunneling effects are neglected. In the current calculation, only the activation energy barrier is considered as a main factor governing the reaction rate.

Results and Discussion

S2. Geometrical parameters

Table S1. Calculated geometrical parameters for complexes **L0** – **L3**.

Complex	θ^{0a}	$<\theta>^b$	σ_1^b	$d^0_{C\text{-}N}{}^a$	$<d_{C\text{-}N}>^b$	σ_2^b
**L0**	66.8	57.7	8.9	1.424	1.413	0.027
**L1**	86.5	74.4	9.2	1.431	1.425	0.031
**L2**	90.5	76.7	9.9	1.432	1.426	0.030
**L3**	91.0	80.5	9.5	1.433	1.433	0.043

[a]Dihedral angle (θ^0 in °) and C–N bond length ($d^0_{C–N}$ in Å) of complexes **L0** – **L3** extracted from the static DFT calculations. [b]Time-averaged dihedral angle ($<\theta>$ in °), C-N bond length ($<d_{C–N}>$ in Å), and corresponding standard deviations of complexes **L0** – **L3** during the free and constrained MD simulations after the photooxidation of NDI dye. See Scheme 1 for the identification of the dihedral angle and the C–N bond.

S3. Molecular orbital and electronic structure

Table S2. Selected frontier molecular orbital energy levels and energy difference between SOMO WOC and SOMO dye (ΔE_{SOMO}, in eV) of complexes **L0 – L3** ($^2([Ru^{IV}=O]_i^{2+}–NDI^{+\bullet})$, *i* = 0 – 3) after the photooxidation of NDI dye.[a]

Intermediate		$^2([Ru^{IV}=O]_i^{2+}–NDI^{+\bullet})$ (*i* = 0 – 3) $\uparrow\uparrow\ \downarrow$			
**Energy level**	**Orbital**	**Energy**			
		**L0**	**L1**	**L2**	**L3**
**LUMO**	α	-6.123	-6.161	-6.177	-6.202
**HOMO** (*SOMO WOC*)	α	-6.275	-6.272	-6.283	-6.299
**HOMO-1** (*SOMO dye*)	β	-6.468	-6.504	-6.520	-6.544
**HOMO-2**	α	-6.732	-6.762	-6.754	-6.787
**HOMO-3**	β	-6.765	-6.795	-6.787	-6.819
**HOMO-4** (*SOMO WOC*)	α	-6.827	-6.806	-6.830	-6.833
**ΔE_{SOMO}**		0.193	0.231	0.237	0.245

[a]The initial geometry of all the complexes **L0** – **L3** are optimized with the ADF program using OPBE functional and the TZP basis set. The continuum solvation model (COSMO) is used to describe the water environment. Only the unpaired electrons are indicated by vertical arrows explicitly (green for unpaired electron localized on the catalyst and blue for unpaired electron on the oxidized $NDI^{+\bullet}$). SOMO represents the singly occupied molecular orbital. All energies are in eV.

S4. Equilibration of the WOC-dye complexes in explicit solvent model

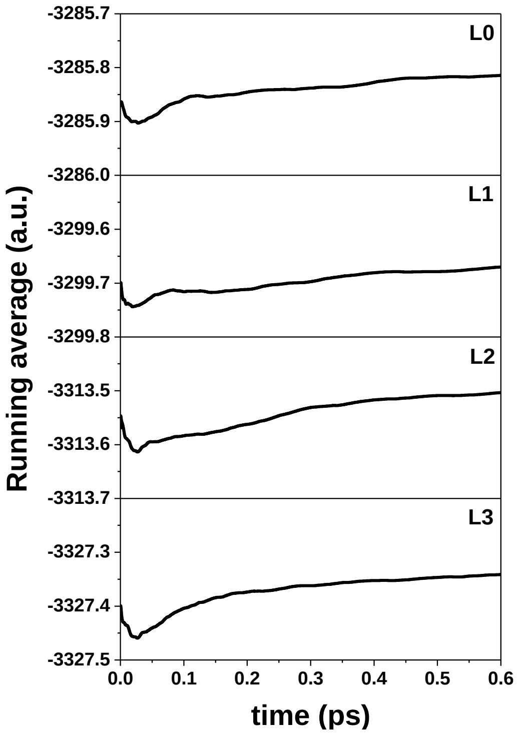

Figure S1. The running average of the Kohn-Sham energy (in a.u.) as a function of time for the equilibration simulation of the photooxidized complexes **L0** – **L3**, respectively. The running average reaches a stable value even within this relatively short MD timescale of ~0.6 ps.

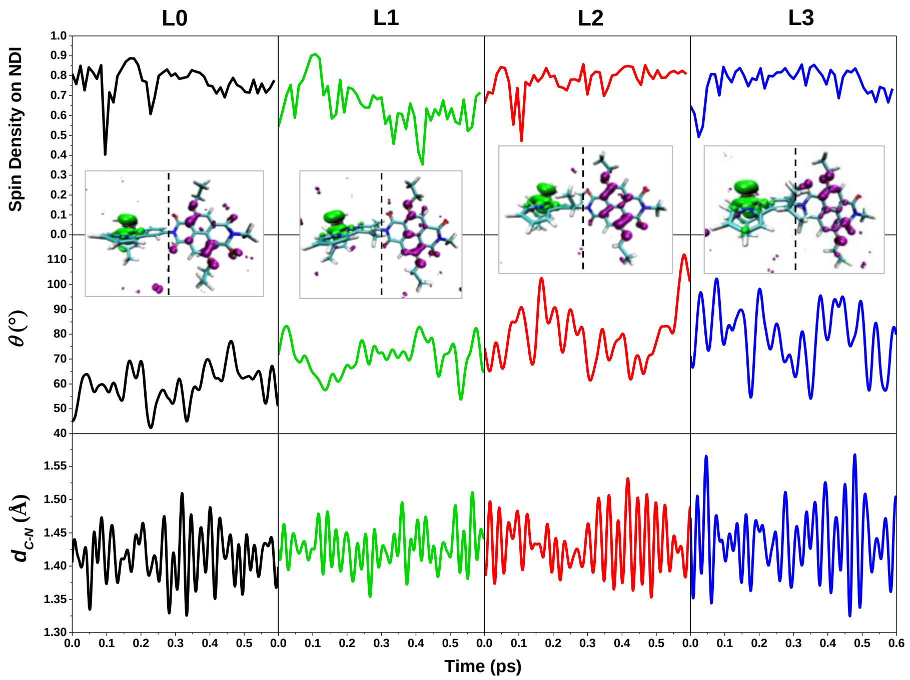

Figure S2. Spin density integrated over half of the simulation box including the NDI dye (right-hand side of the dashed black line in the insets), time evolution of the dihedral angle (θ) and C–N bond length ($d_{C–N}$) of complexes **L0** – **L3** (^{2}([Ru^{IV}=O]$_i^{2+}$–NDI$^{+\bullet}$), i = 0 – 3) along the free MD trajectories after the photooxidation of NDI dye. The insets show the spin density isosurface computed at a snapshot taken at the end of each free MD simulation of complexes **L0** – **L3**, respectively, in the doublet state with two unpaired α electrons localized on the catalyst (green spin density isosurface) and one unpaired β electron on the NDI dye (purple spin density isosurface). An integrated spin density value of 1 corresponds to one unpaired β electron ($\downarrow$).

S5. Continued constrained 2.5 Å MD simulation for L1

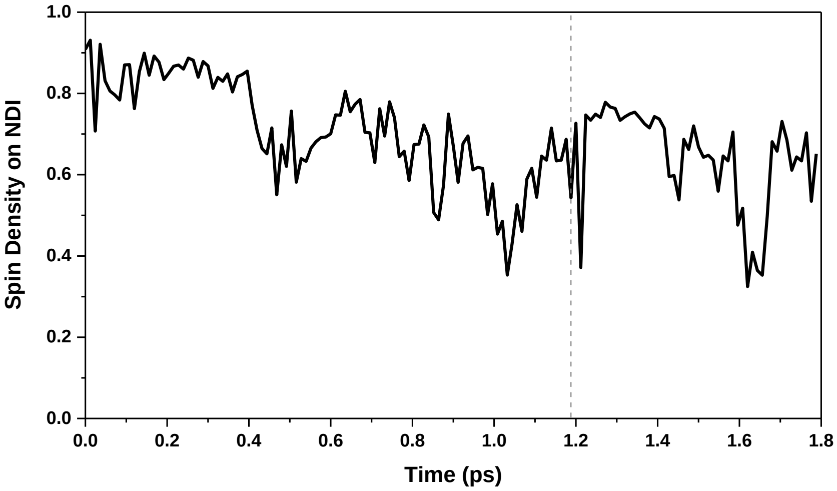

Figure S3. Spin density integrated over half of the simulation box including the NDI dye of complex **L1** along the longer constrained 2.5 Å MD trajectory. This figure clearly shows that the spin density fluctuates considerably during the overall constrained 2.5 Å MD simulation, indicating that the electron transfers back and forth between the WOC and dye. In other words, the electron transfer is not completed in such a MD timescale at the current stage of the reaction coordinate d(O_i←O_{ii}) = 2.5 Å.

S6. Running average of the constraint force as a function of time

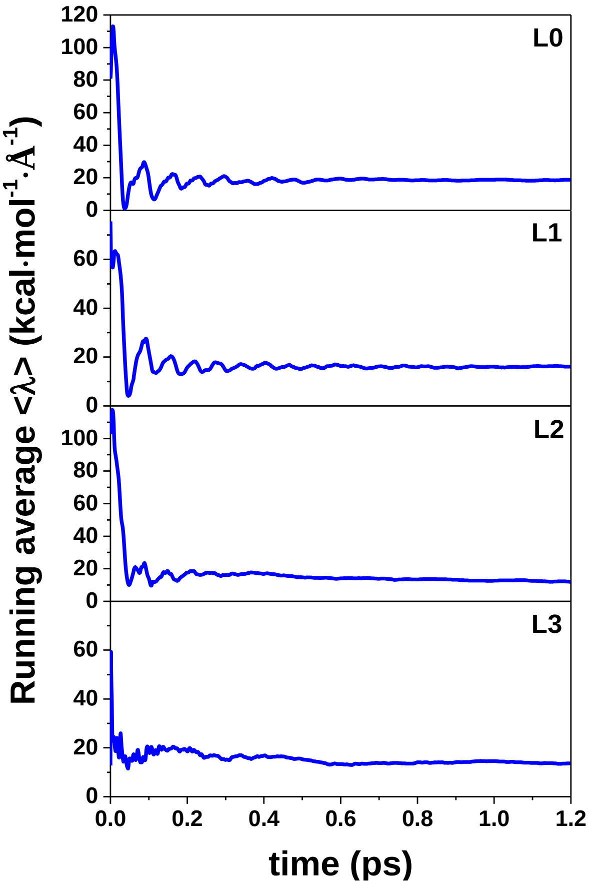

Figure S4. The running average of the constraint force (<λ>, in kcal mol^{-1} $Å^{-1}$) as a function of time for the distance constraint d(O_i←O_{ii}) = 2.5 Å of complexes **L0** – **L3**, respectively. The running average reaches a stable value within this MD timescale of ~1.2 ps.

S7. Continued constrained 1.8 Å MD simulation for L3

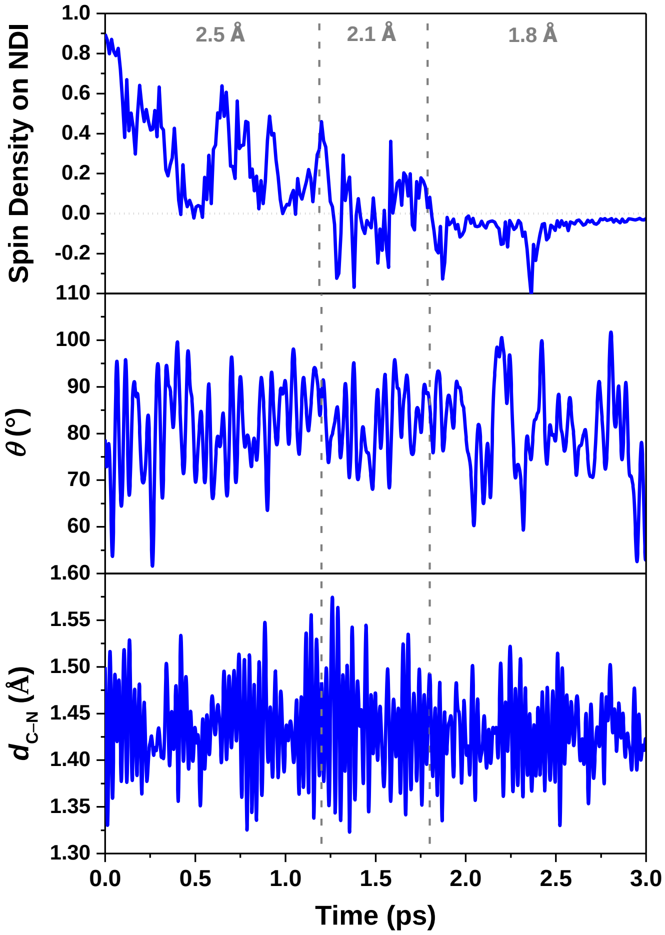

Figure S5. Spin density integrated over half of the simulation box including the NDI dye, time evolution of the dihedral angle (θ) and C–N bond length ($d_{C–N}$) of complex **L3** only along the constrained MD trajectories after the photooxidation of NDI dye. The value of the constrained reaction coordinate d(O_i←O_{ii}) in the MD simulations is noted in grey.

S8. Proton transfer for L0 – L3 at d(O_i←O_{ii}) = 1.8 Å

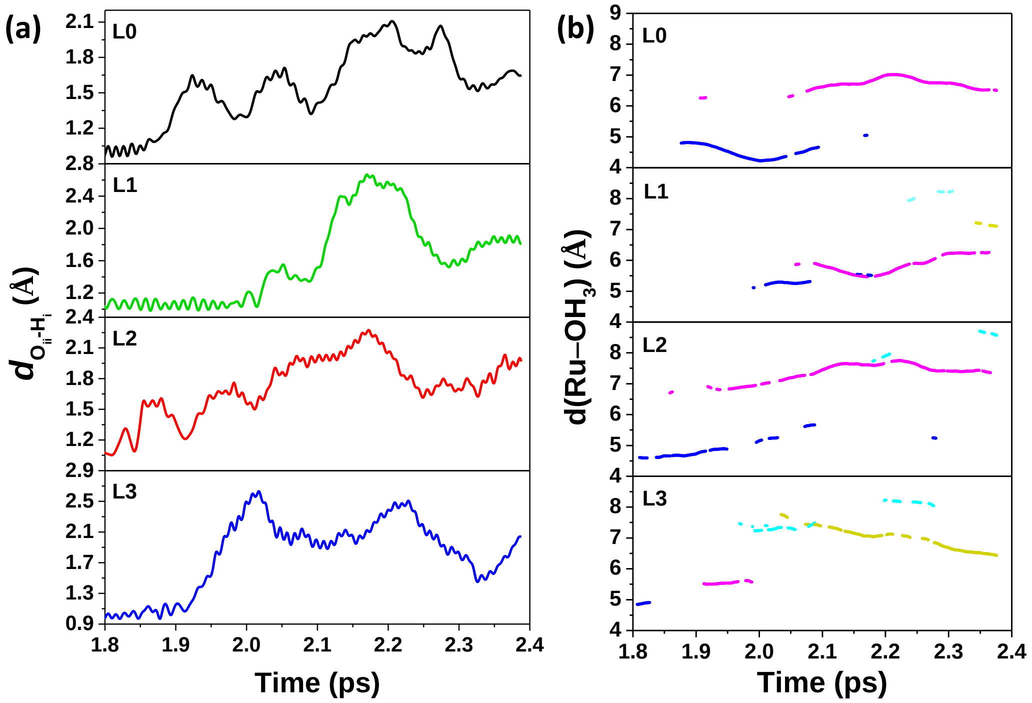

Figure S6. (a) Time evolution of the geometrical parameter $d_{O_{ii}-H_i}$ along the constrained MD trajectory with d(O_i←O_{ii}) = 1.8 Å for complexes **L0** – **L3**. (b) The distance between Ru and H_3O^+, defined as an oxygen atom with 3 H within a radius of 1.2 Å, illustrating the proton diffusion during the constrained 1.8 Å MD simulations for complexes **L0** – **L3**. The analysis of the trajectories shows that only one oxygen is in the H_3O^+ form at any time, and the excess proton associates primarily to 2 – 4 different oxygens (indicated with different colours) during the simulation. This figure clearly shows that the proton diffuses from the first solvation shell of the ruthenium center to the second or even third and fourth solvation shell rapidly within this relatively short MD timescale of ~0.6 ps. The time range is consistent with that in **Figure 3**.

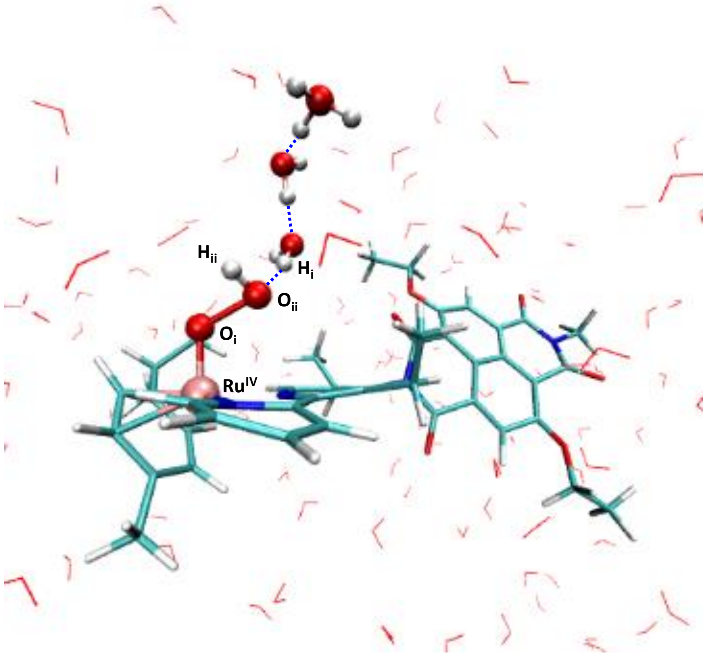

Figure S7. Snapshot taken at the end of the constrained 1.8 Å simulation for complex **L2**. The attacking water molecule and the neighboring water molecules forming the hydrogen-bonded chain are represented with ball & stick. The dashed blue lines indicate the hydrogen bonds. This figure clearly shows that the proton H_i has been totally released by the attacking water molecule and diffuses rapidly into the solvent bulk via a “chain” of hydrogen-bonded water molecules even within the MD timescale of ~0.6 ps.

S9. Constrained 1.6 Å MD simulation

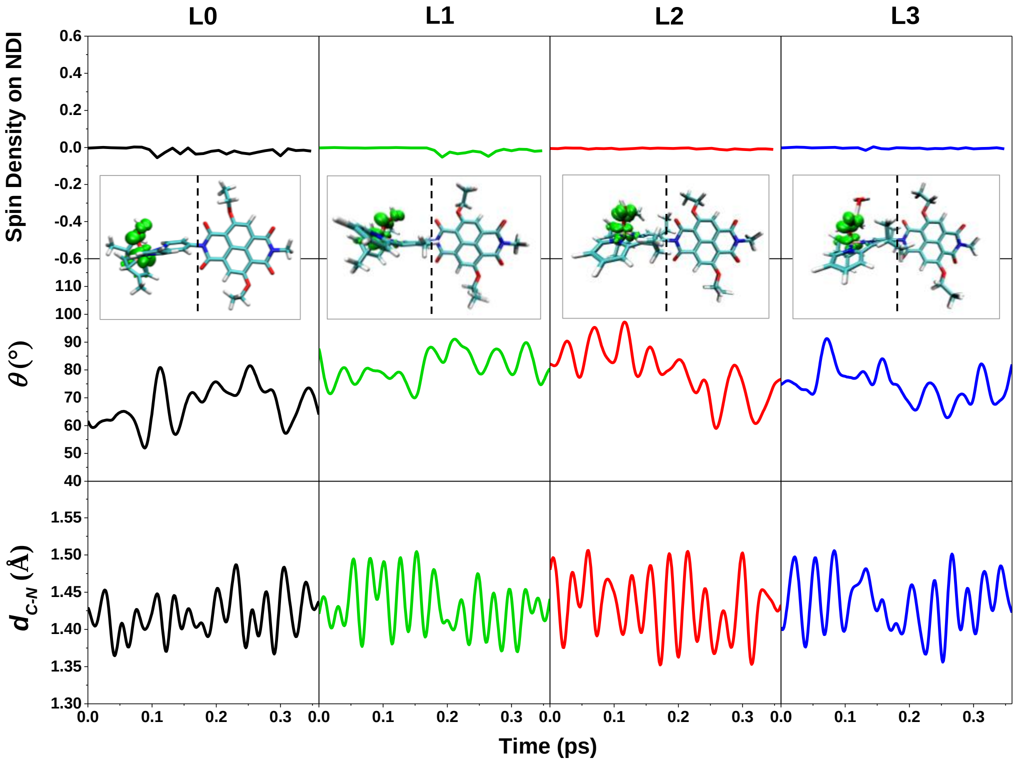

Figure S8. Spin density integrated over half of the simulation box including the NDI dye (right-hand side of the dashed black line in the insets), time evolution of the dihedral angle (θ) and C–N bond length (d_{C-N}) of complexes **L0** – **L3** along the constrained 1.6 Å MD trajectories after the PCET process. The insets show the spin density isosurface computed at a snapshot taken at the end of each MD simulation of complexes **L0** – **L3**, respectively, in the doublet state with one unpaired α electron localized on the catalyst (green spin density isosurface) and no unpaired β electron on the NDI dye. An integrated spin density value of -1 corresponds to one unpaired α electron (↿).

S10. O−O bond formation during the free MD simulation

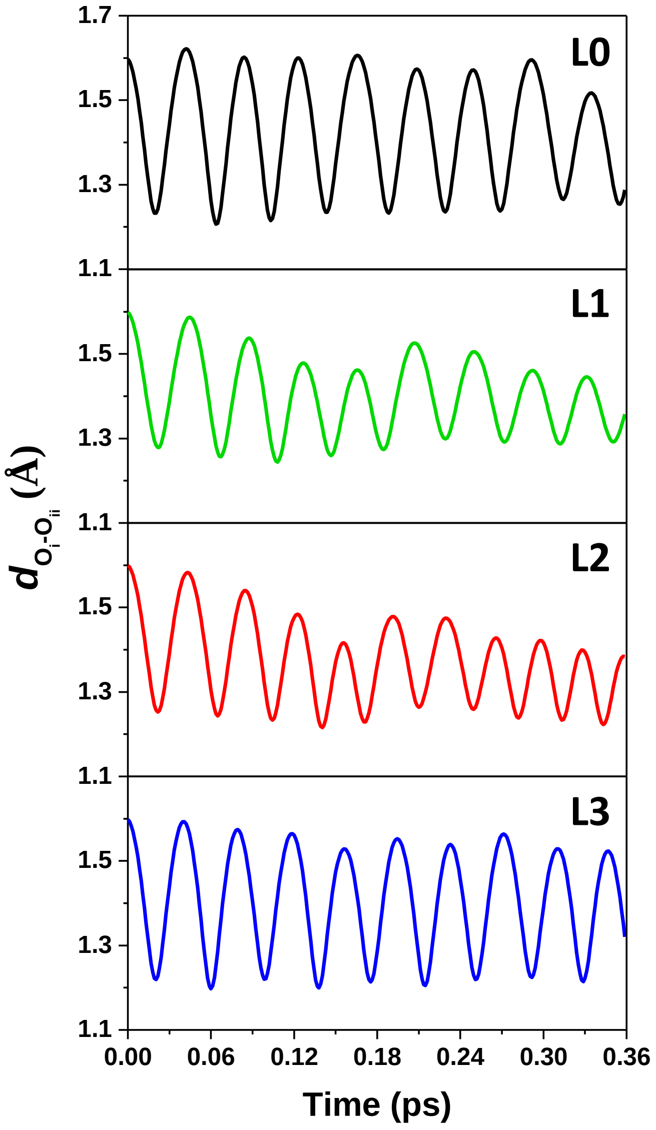

Figure S9. Time evolution of the distance between two oxygen atoms O_i and O_{ii} ($d_{O_i-O_{ii}}$) along the free MD trajectory after the release of constraint at the end of the constrained 1.6 Å MD simulation of complexes **L0** – **L3**.

S11. Time-averaged constraint force and standard deviation

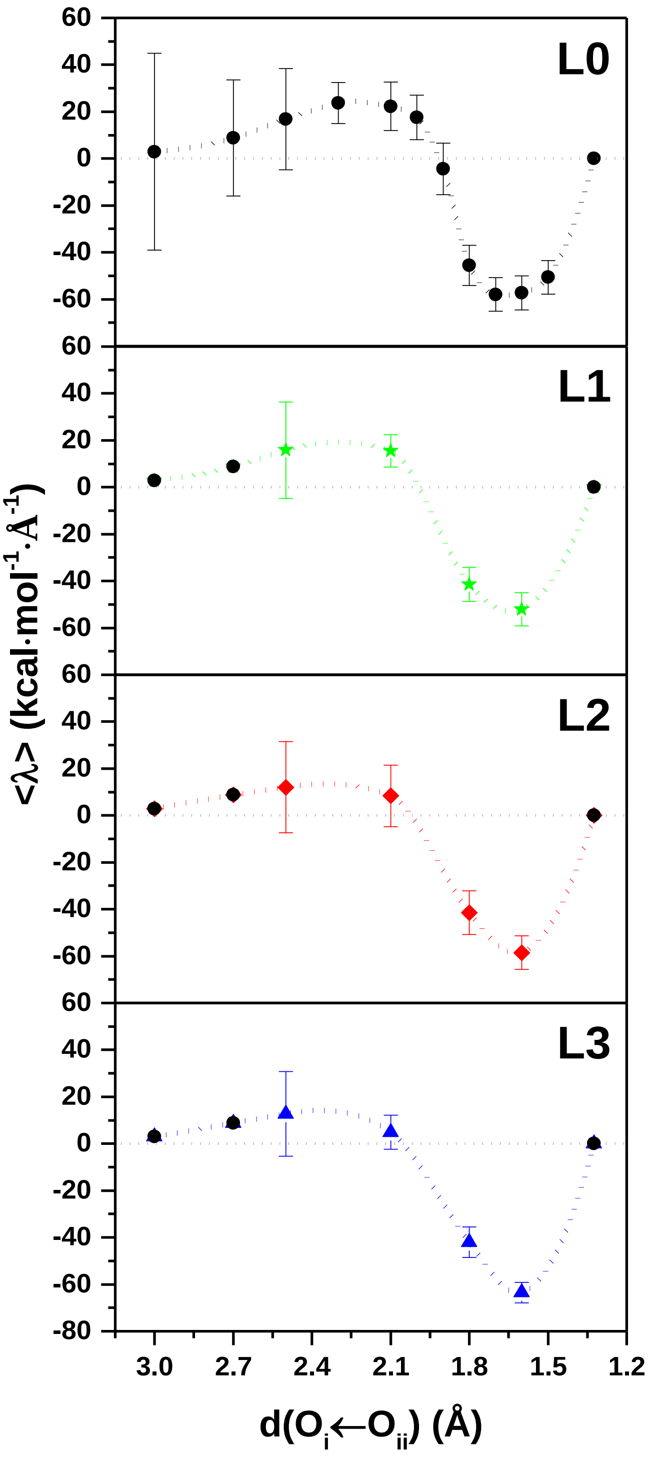

Figure S10. Time-averaged constraint force represented by the Lagrangian multiplier <λ> computed for each constrained MD simulation as a function of the reaction coordinate d(O_i←O_{ii}) for complexes **L0** – **L3**. The Akima splines (100 points) is used to interpolate the mean forces. The error bars indicate the standard deviations.

S12. Fourier transform and vibrational density of states (VDOS)

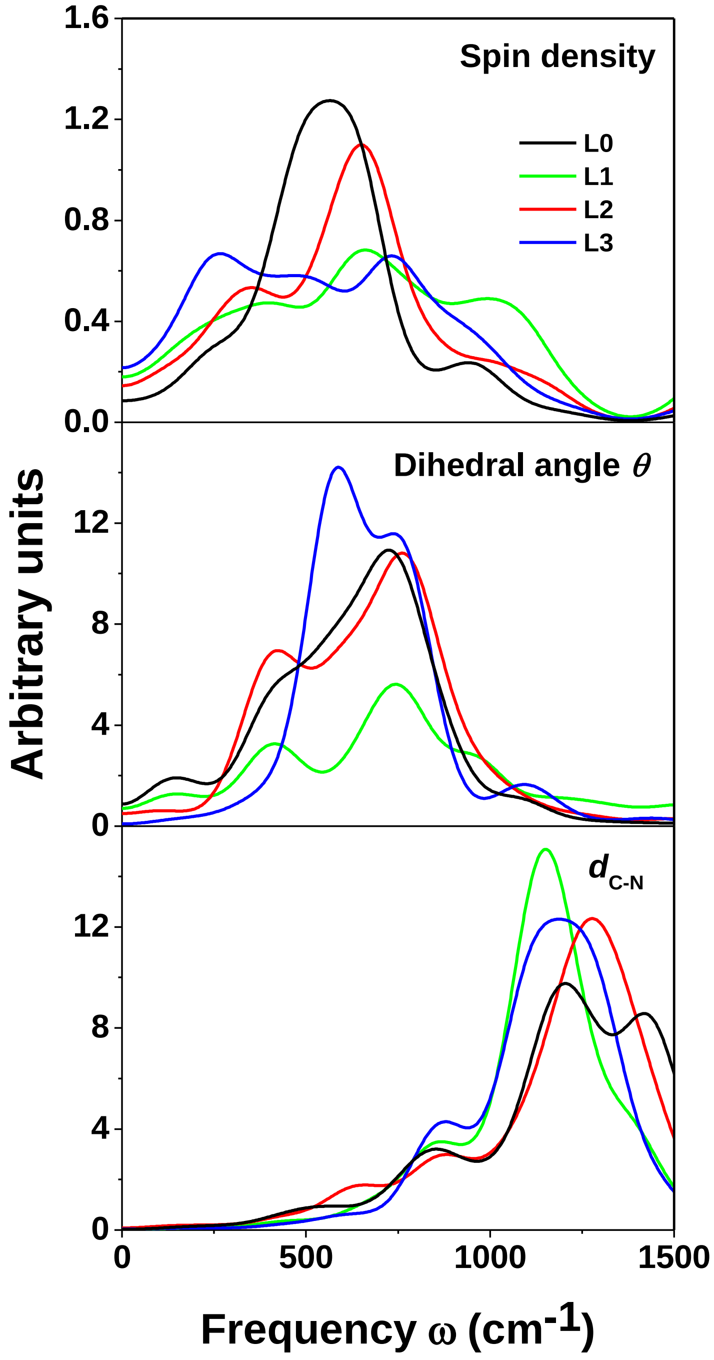

Figure S11. Frequency spectrum associated to the electron transfer (top) and the vibrational density of states (VDOS) of the dihedral angle (θ, middle) and the C-N bond length ($d_{C\text{-}N}$, bottom) for complexes **L0** – **L3** extracted from the constrained 2.5 and 2.1 Å MD trajectories corresponding to **Figure 3**.

S13. Constrained MD simulation with fixed dihedral angle *θ* for L3

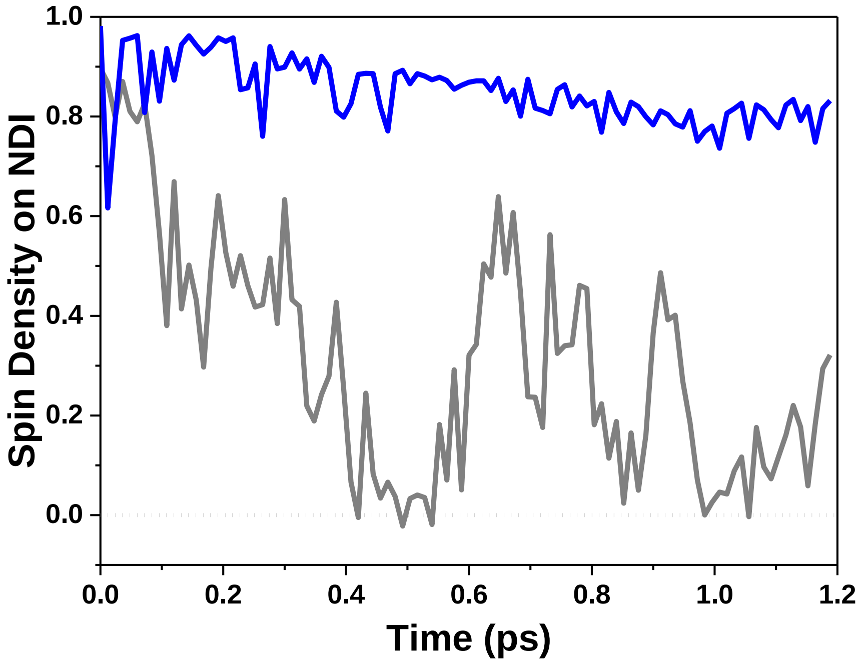

Figure S12. Spin density integrated over half of the simulation box including the NDI dye of complex **L3** along the constrained 2.5 Å MD trajectory with fixed dihedral angle θ = 91° after the photooxidation of NDI dye (blue line). Before this constrained MD simulation, a simulation of ~0.36 ps with fixed dihedral angle θ = 91° was performed to equilibrate the solvated system. The data without constraint on the dihedral angle θ of **L3** (grey line) is also presented for comparison, which is extracted from **Figure 3**.

S14. Geometry optimization after PCET

Table S3. Calculated geometrical parameters of the initial and final intermediates after geometry optimization for complexes **L0** – **L3**.

Complex	Intermediate			
	Ru=O[a]		Ru–OOH[b]	
	$\theta_{\_ini}$	$d_{C\text{-}N\_ini}$	$\theta_{\_fin}$	$d_{C\text{-}N\_fin}$
**L0**	66.8	1.424	66.5	1.422
**L1**	86.5	1.431	86.7	1.428
**L2**	90.5	1.432	90.3	1.428
**L3**	91.0	1.433	90.3	1.428

[a]Dihedral angle ($\theta_{\_ini}$ in °) and C–N bond length ($d_{C–N\_ini}$ in Å) of the initial intermediate (Ru=O) after geometry optimization for complexes **L0** – **L3** extracted from the static DFT calculations. [b]Dihedral angle ($\theta_{\_fin}$ in °) and C–N bond length ($d_{C–N\_fin}$ in Å) of the final intermediate (Ru–OOH) after the third PCET step for complexes **L0** – **L3** extracted from the static DFT calculations. See **Scheme 1** for the atomic labeling.

S15. TDDFT calculation of the excitation energies near the transition state

Table S4. Excitation energy (E, kcal mol^{-1}), oscillator strengths (*f*), and related molecular orbitals for the first excitation of **L0** – **L3** together with the attacking water molecule.[a]

Complex	d(O_i–O_{ii})	E	*f*	HOMO	LUMO
**L0**	1.9	1.788	0.011	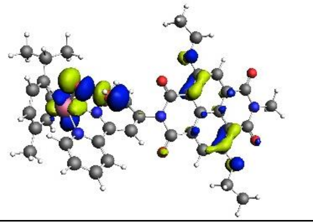	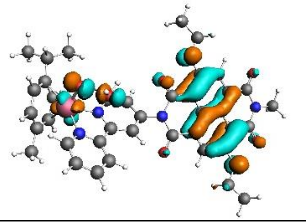
**L1**	2.0	1.646	0.009	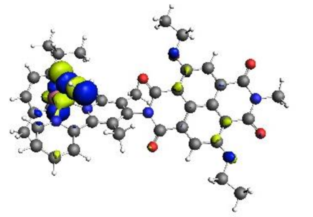	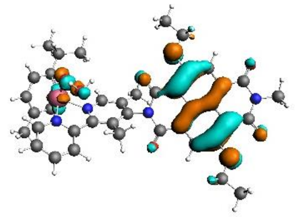
**L2**	2.0	1.307	0.006	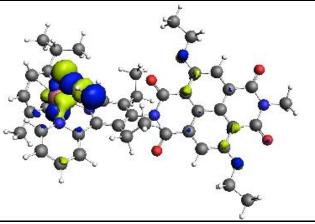	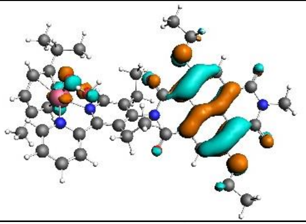
**L3**	2.0	1.291	0.005	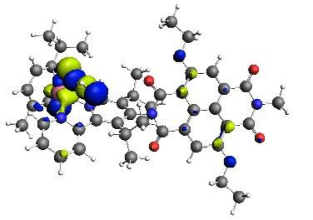	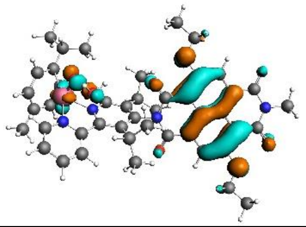

[a]The geometry of all the complexes **L0** – **L3** together with the attacking water molecule are firstly optimized with the ADF program using OPBE functional and the TZP basis set. The TD-DFT calculations are then performed at the same lavel. The continuum solvation model (COSMO) is used to describe the water environment. The distance between the two oxygen atoms O_i and O_{ii} (d(O_i–O_{ii}), Å) is fixed to certain values which is taken around the transition state according to **Figure 4**. The first excitation is mainly related to the transition from HOMO (SOMO WOC) to LUMO (SOMO dye). SOMO represents the singly occupied molecular orbital.

References

[1] M. Swart, A. W. Ehlers, K. Lammertsma, *Mol. Phys.* **2004**, *102*, 2467-2474.

[2] A. Monti, J. M. de Ruiter, H. J. M. de Groot, F. Buda, *J. Phys. Chem. C* **2016**, *120*, 23074-23082.

[3] a) A. T. P. Carvalho, M. Swart, *J. Chem. Inf. Model.* **2014**, *54*, 613-620; b) A. R. Groenhof, A. W. Ehlers, K. Lammertsma, *J. Am. Chem. Soc.* **2007**, *129*, 6204-6209; c) J. Conradie, A. Ghosh, *J. Chem. Theory and Comput.* **2007**, *3*, 689-702; d) J. L. Vallés-Pardo, M. C. Guijt, M. Iannuzzi, K. S. Joya, H. J. M. de Groot, F. Buda, *ChemPhysChem* **2012**, *13*, 140-146.

[4] a) A. Klamt, *J. Phys. Chem.* **1995**, *99*, 2224-2235; b) A. Klamt, V. Jonas, *J. Chem. Phys.* **1996**, *105*, 9972-9981.

[5] a) G. te Velde, F. M. Bickelhaupt, E. J. Baerends, C. Fonseca Guerra, S. J. A. van Gisbergen, J. G. Snijders, T. Ziegler, *J. Comput. Chem.* **2001**, *22*, 931-967; b) ADF2017, SCM, Theoretical Chemistry, Vrije Universiteit, Amsterdam, The Netherlands, http://www.scm.com.

[6] Accelrys Software Inc. *Discovery Studio Modeling Environment*, Accelrys Software Inc.: San Diego, 2012

[7] B. R. Brooks, R. E. Bruccoleri, B. D. Olafson, D. J. States, S. Swaminathan, M. Karplus, *J. Comput. Chem.* **1983**, *4*, 187-217.

[8] a) G. Ciccotti, M. Ferrario, *Mol. Simul.* **2004**, *30*, 787-793; b) B. Ensing, E. J. Meijer, P. E. Blöchl, E. J. Baerends, *J. Phys. Chem. A* **2001**, *105*, 3300-3310; c) F. Costanzo, R. G. Della Valle, *J. Phys. Chem. B* **2008**, *112*, 12783-12789.

[9] Y. Shao, J. M. de Ruiter, H. J. M. de Groot, F. Buda, *J. Phys. Chem. C* **2019**, *123*, 21403-21414.

[10] a) W. K. d. Otter, W. J. Briels, *J. Chem. Phys.* **1998**, *109*, 4139-4146; b) M. Sprik, G. Ciccotti, *J. Chem. Phys.* **1998**, *109*, 7737-7744; c) L. Bernasconi, E. J. Baerends, M. Sprik, *J. Phys. Chem. B* **2006**, *110*, 11444-11453; d) L. Bernasconi, A. Kazaryan, P. Belanzoni, E. J. Baerends, *ACS Catal.* **2017**, *7*, 4018-4025.

[11] a) W. Humphrey, A. Dalke, K. Schulten, *J. Mol. Graphics* **1996**, *14*, 33-38; b) VMD - Visual Molecular Dynamics. *Theoretical Chemistry and Computational Biophysics Group*, University Of Illinois: Urbana, 2016.

[12] a) H. Eyring, *J. Chem. Phys.* **1935**, *3*, 107-115; b) K. J. Laidler, M. C. King, *J. Phys. Chem.* **1983**, *87*, 2657-2664; c) E. Pollak, P. Talkner, *Chaos* **2005**, *15*, 026116.
